# HbA1c Variability and Cardiovascular Events in Patients with Prostate Cancer Receiving Androgen Deprivation Therapy

**DOI:** 10.1016/j.euros.2022.11.002

**Published:** 2022-12-15

**Authors:** Jeffrey Shi Kai Chan, Yan Hiu Athena Lee, Kang Liu, Jeremy Man Ho Hui, Edward Christopher Dee, Kenrick Ng, Danish Iltaf Satti, Tong Liu, Gary Tse, Chi Fai Ng

**Affiliations:** a Cardio-Oncology Research Unit, Cardiovascular Analytics Group, Hong Kong, China; b Division of Urology, Department of Surgery, Faculty of Medicine, The Chinese University of Hong Kong, Hong Kong, China; c Department of Radiation Oncology, Memorial Sloan Kettering Cancer Center, New York, NY, USA; d Department of Medical Oncology, University College London Hospitals NHS Foundation Trust, London, UK; e Tianjin Key Laboratory of Ionic-Molecular Function of Cardiovascular Disease, Department of Cardiology, Tianjin Institute of Cardiology, Second Hospital of Tianjin Medical University, Tianjin, China; f Kent and Medway Medical School, Canterbury, Kent, UK; g SH Ho Urology Centre, The Chinese University of Hong Kong, Hong Kong, China

**Keywords:** Cardio-oncology, Androgen deprivation therapy, Prostate cancer, Major adverse cardiovascular events

## Abstract

**Background:**

Androgen deprivation therapy (ADT) worsens glycaemic control and cardiovascular outcomes. The prognostic value of visit-to-visit HbA1c variability (VVHV) has been unexplored in prostate cancer (PCa) patients receiving ADT.

**Objective:**

To explore the effect of ADT on VVHV and the cardiovascular prognostic value of VVHV.

**Design, setting, and participants:**

PCa patients receiving ADT in Hong Kong between January 1, 1993 and March 31, 2021 were included in this retrospective cohort study. Those with fewer than three HbA1c results available within 3 yr after ADT initiation, <6 mo of ADT, missing baseline HbA1c, prior diagnosis of any component of major adverse cardiovascular events (MACEs), and MACEs occurring within 3 yr were excluded. Patients were followed up until September 31, 2021.

**Outcome measurements and statistical analysis:**

The outcome was MACEs (composite of heart failure, myocardial infarction, stroke, and cardiovascular mortality). VVHV was calculated from HbA1c levels within 3 yr after and, separately where available, before ADT initiation using coefficient of variation (CV; standard deviation [SD] divided by mean) and average real variability (ARV; average difference between consecutive measurements).

**Results and limitations:**

Altogether, 1065 patients were analysed (median age 74.4 yr old [interquartile range 68.3–79.5 yr]). In 709 patients with VVHV available before and after ADT initiation, VVHV increased after ADT initiation (*p* < 0.001), with 473 (66.2%) and 474 (66.9%) having increased CV and ARV, respectively. Over a median follow-up of 4.3 yr (2.8–6.7 yr), higher VVHV was associated with a higher risk of MACEs (adjusted hazard ratio [per SD] for CV 1.21 [95% confidence interval: 1.02, 1.43], *p* = 0.029; ARV 1.25 [1.06, 1.48], *p* = 0.008). Limitations included residual confounding and selection bias.

**Conclusions:**

In PCa patients receiving ADT, VVHV increased after ADT initiation. Higher VVHV was associated with an increased risk of MACEs.

**Patient summary:**

In prostate cancer patients receiving androgen deprivation therapy (ADT), glycaemic control is less stable after initiating ADT, which was associated with an increased cardiovascular risk.

## Take Home Message

In patients with prostate cancer receiving androgen deprivation therapy (ADT), visit-to-visit HbA1c variability (VVHV) increased after ADT initiation and higher VVHV was associated with higher cardiovascular risks. VVHV is a potential tool for cardiovascular risk stratification in these patients.

## 1 Introduction

Prostate cancer (PCa) was the third most common cancer globally in 2020, with 1.4 million incident cases and accounting for over 375 000 deaths [1]. Androgen deprivation therapy (ADT) is one of the key therapies for PCa, in which testosterone activity is suppressed pharmacologically and/or surgically [2], [3]. ADT is recommended alone or in combination with other therapeutic modalities for diseases of intermediate or higher risk [3], [4]. Despite its established oncological efficacy, studies have shown associations between ADT and increased risks of diabetes mellitus (DM), cardiovascular mortality, myocardial infarction (MI), and stroke [5], [6], [7], and among diabetic patients, worsened diabetic control and a higher risk of diabetic complications [8], [9], [10]. Currently, risk factors and prognosticators for adverse cardiovascular events among patients with PCa receiving ADT remain actively investigated.

Studies of the glycaemic effects of ADT focused on glycaemic markers, such as HbA1c, as point estimates at fixed time points [8], [9], [10]. Emerging evidence suggested that visit-to-visit HbA1c variability (VVHV) has incremental prognostic value atop point estimates that neglect longitudinal variations in HbA1c levels. Higher VVHV, reflecting more fluctuating HbA1c levels between hospital or clinic visits (ie, less stable glycaemic control), has been associated with increased risks of mortality and adverse cardiovascular events in patients with and without DM [11], [12], [13], [14], [15]. Nonetheless, the effect of ADT on VVHV, as well as the prognostic value of VVHV in patients with PCa receiving ADT, is unexplored. Given the adverse cardiometabolic effects of ADT and the prognostic value of VVHV, this study aimed to test the hypothesis that ADT adversely affects VVHV, and that VVHV is prognostic of cardiovascular outcomes in patients with PCa receiving ADT.

## 2 Patients and methods

This retrospective cohort study was approved by Joint Chinese University of Hong Kong—New Territories East Cluster Clinical Research Ethics Committee, and was conducted according to the Declaration of Helsinki and the Strengthening the Reporting of Observational Studies in Epidemiology guideline. As only retrospective, deidentified data were used, the need for individual consent was waived.

### 2.1 Source of data

Data were acquired from the Clinical Data Analysis and Reporting System (CDARS), a population-based administrative database recording basic demographics, diagnoses, laboratory tests, medication prescriptions, and medical procedures of all patients attending public hospitals and clinics in Hong Kong, which cover the entire Hong Kong and serve 90% of the population [16]. CDARS encodes diagnoses using the International Classification of Diseases, ninth revision (ICD-9) codes regardless of the time of data input, as ICD-10 codes have not been implemented in CDARS to date. Mortality data were acquired from the linked Hong Kong Death Registry, a governmental registry of mortality data for Hong Kong citizens. CDARS and the linked Hong Kong Death Registry have been used extensively in research, and have been demonstrated to have good coding accuracy and data completeness [17], [18], [19], [20], [21], [22].

### 2.2 Patient population

Adult (aged 18 yr or above) patients with PCa who received ADT between January 1, 1993 and March 31, 2021 were retrospectively identified and included. ADT included medical castration (leuprorelin, triptorelin, goserelin, or degarelix; other gonadotropin-releasing hormone agonists and antagonists were not available in Hong Kong during the study period) and bilateral orchiectomy (BO). Patients with fewer than three HbA1c measurements available within 3 yr after ADT initiation, <6 mo of ADT, missing baseline HbA1c level (within 3 yr prior to ADT initiation), known heart failure (HF), MI, or stroke, and those with the primary outcome occurring within 3 yr were excluded. Baseline covariates recorded are summarised in the Supplementary material.

### 2.3 Follow-up and outcomes

All patients were followed up from the date of ADT initiation until September 31, 2021. The primary outcome was major adverse cardiovascular events (MACEs), a composite of HF, MI, stroke, and cardiovascular mortality, which occurred at least 3 yr after the initiation of ADT. HF, MI, and stroke were identified by ICD-9 codes listed in Supplementary Table 1, whilst cardiovascular mortality was identified by ICD-9 or ICD-10 codes listed in Supplementary Table 2.

### 2.4 Statistical analyses

VVHV was calculated using all HbA1c measurements within 3 yr after ADT initiation. For patients with at least three HbA1c measurements available within 3 yr before ADT initiation, mean HbA1c and VVHV prior to ADT initiation were also calculated; no imputations were performed for patients with fewer than three HbA1c measurements within 3 yr prior to ADT initiation. VVHV was measured by the coefficient of variation (CV; $\frac{\mathrm{standard}\;deviation}{\mathrm{mean}}$), and average real variability (ARV; $\frac{\sum_{k=1}^{N-1}\left|HbA1c_{k+1}-HbA1c_{k}\right|}{N-1}$, where *N* is the number of HbA1c measurements available, and *k* ranges from 1 to *N* – 1) [23], [24]. For patients who had at least three HbA1c measurements available within 3 yr prior to ADT initiation, per-unit change in VVHV was defined as $\left[Post-ADT\;VVLV\right]-\left[Pre-ADT\;VVLV\right]$ and the percentage change was defined as $\frac{\left[Post-ADT\;\;VVLV\right]-\left[Pre-ADT\;\;VVLV\right]}{\left[Pre-ADT\;\;VVLV\right]}\times 100\%$.

Details of statistical analyses are summarised in the Supplementary material. Briefly, in those with at least three HbA1c measurements available within 3 yr prior to ADT initiation, pre- and post-ADT VVHV values were compared. The proportional hazard assumption was not violated when tested using Schoenfield residuals. Multivariable Cox regression adjusted for significant baseline confounders identified by univariable Cox regression was used to evaluate the prognostic value of VVHV, with hazard ratios representing per standard deviation (SD) increase in VVHV measures. VVHV was also analysed as quartiles. Three a priori subgroup analyses were performed for both the changes in VVHV and the prognostic value of VVHV by (1) diagnosis of DM, (2) use of antidiabetic medication(s), and (3) types of ADT.

Sensitivity analyses were performed for the evaluation of the prognostic value of VVHV. An a priori sensitivity analysis was performed using multivariable competing risk regression with noncardiovascular mortality as the competing event. A post hoc sensitivity analysis was performed where differences in restricted mean survival time were used to compare between groups. A second post hoc sensitivity analysis was performed where only patients with at least 1 yr of ADT were analysed. All *p* values were two sided, with *p* < 0.05 considered statistically significant.

## 3 Results

**Fig. 1:**
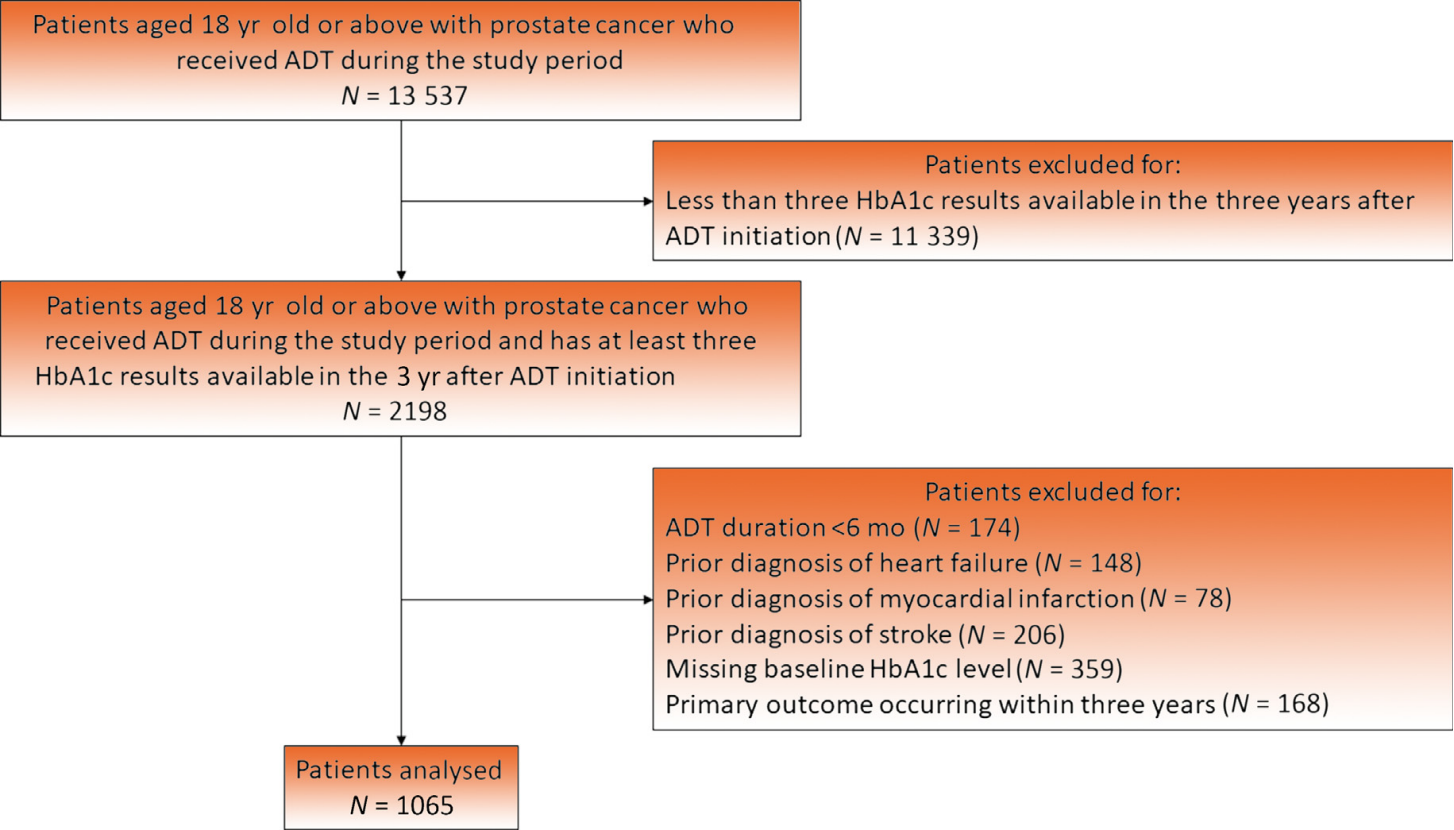
Study flowchart. ADT = androgen deprivation therapy.

Altogether, 13 537 patients were identified, of whom 2198 had at least three HbA1c results available within 3 yr after ADT initiation. After applying the exclusion criteria, 1065 patients were included (median age 74.4 yr [interquartile range 68.3–79.5 yr]; Fig. 1), of whom 850 (79.8%) had DM. Characteristics of the included patients are summarised in Table 1. Within the 3 yr after ADT initiation, the patients had a median of five (four to six) available HbA1c measurements. The median CV of HbA1c was 0.081 (0.046–0.135), and the median ARV was 0.57% (0.31–1.03%). The 25th percentile, median, and 75th percentile values were also used for defining the cut-off values for categorising the VVHV markers into quartiles.

**Table 1:** Baseline characteristics of included patients

Total number of patients (*N*)	1065
Age, yr (IQR)	74.4 (68.3–79.5)
Medical castration, *N* (%)	762 (71.6)
Bilateral orchiectomy, *N* (%)	430 (40.4)
ADT duration, yr (IQR)	3.4 (2.3–5.7)
Hypertension, *N* (%)	401 (37.7)
Diabetes mellitus, *N* (%)	850 (79.8)
Dyslipidaemia, *N* (%)	166 (15.6)
Ischaemic heart disease, *N* (%)	155 (14.6)
Chronic kidney disease, *N* (%)	41 (3.9)
Atrial fibrillation, *N* (%)	37 (3.5)
Known malignancy, *N* (%)	103 (9.7)
Prior radiotherapy, N (%)	41 (3.9)
Prior radical prostatectomy, *N* (%)	269 (25.3)
ACEI/ARB use, *N* (%)	599 (56.2)
Beta-blocker use, *N* (%)	476 (44.7)
Metformin use, *N* (%)	627 (58.9)
Sulphonylurea use, *N* (%)	565 (53.1)
Insulin use, *N* (%)	183 (17.2)
Dihydropyridine CCB use, *N* (%)	623 (58.5)
Antiplatelet use, *N* (%)	298 (28.0)
Anticoagulant use, *N* (%)	36 (3.4)
Chemotherapy use, *N* (%)	6 (0.6)
Steroid use, *N* (%)	164 (15.4)
HbA1c, % (IQR)	6.7 (6.1–7.4)

ACEI = angiotensin-converting enzyme inhibitor; ADT = androgen deprivation therapy; ARB = angiotensin receptor blocker; CCB = calcium channel blocker; IQR = interquartile range.

### 3.1 Change in VVHV after ADT initiation

Seven hundred and nine patients (66.6%) had at least three HbA1c measurements within the 3 yr prior to ADT initiation, with a median of five (three to seven) measurements in the 3 yr prior to ADT initiation, and five (four to seven) measurements in the 3 yr after ADT initiation.

VVHV increased significantly after ADT initiation, as measured by both CV (0.059 [0.036–0.103] before ADT vs 0.089 [0.054, 0.139] after ADT, *p* < 0.001) and ARV (0.44% [0.26–0.77%] before ADT vs 0.63% [0.39–1.08%] after ADT, *p* < 0.001). The median per-unit change in CV was 0.023 (–0.013 to 0.071), and the median percentage change was 43.0% (–17.3% to 147.5%). The median per-unit change in ARV was 0.17% (–0.09% to 0.50%), and the median percentage change was 43.1% (–18.0% to 138.1%). In total, 473 (66.2%) and 474 (66.9%) had increased CV and ARV of HbA1c after initiating ADT, respectively.

A subgroup analysis by prior diagnosis of DM (Supplementary Table 4), prior use of antidiabetic medication(s) (Supplementary Table 5), and type of ADT (Supplementary Table 6) found that there were generally no differences between subgroups in the change in VVHV after ADT initiation, except for the percentage change in CV of HbA1c, which was significantly smaller in those who used antidiabetic medication(s) than in those who did not use such medication(s) at baseline (*p* = 0.025). There was also a numerical trend for a smaller percentage change in the ARV of HbA1c in those who used antidiabetic medication(s), which approached, but did not reach, statistical significance (*p* = 0.072).

### 3.2 Prognostic value of VVHV

**Fig. 2:**
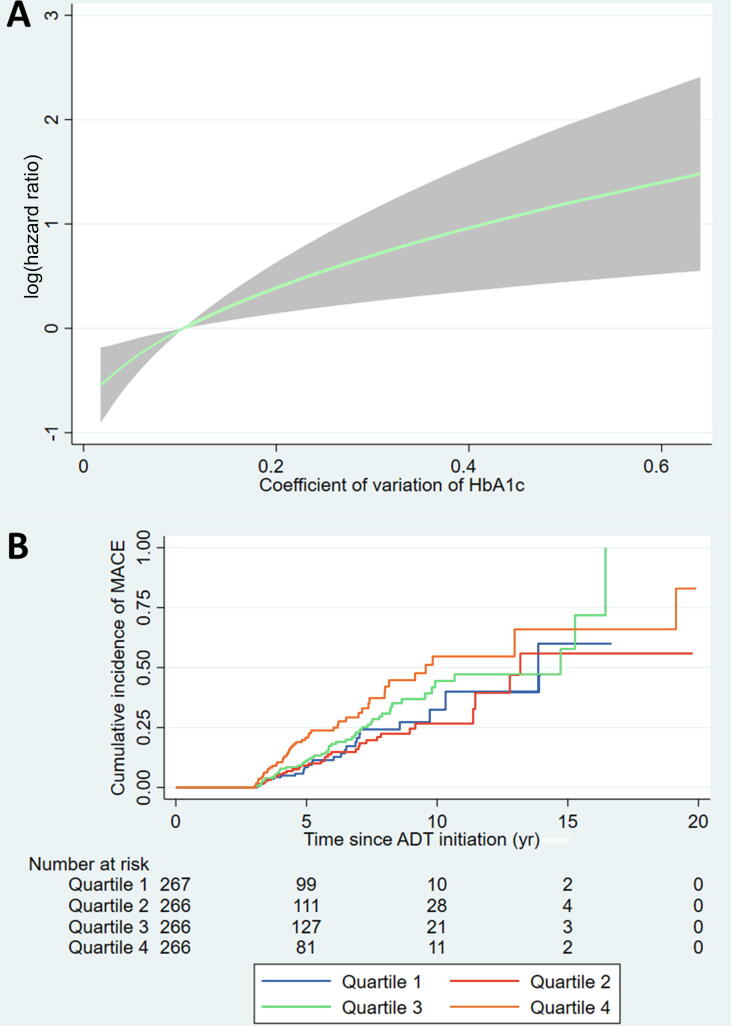
(A) Fractional polynomial plot showing the association between the coefficient of variation (CV) of HbA1c and the risk of major adverse cardiovascular events (MACEs) across the observed range of HbA1c CV. (B) Kaplan-Meier curves showing the cumulative incidence of MACEs in patients in each quartile of the CV of HbA1c. ADT = androgen deprivation therapy.

**Fig. 3:**
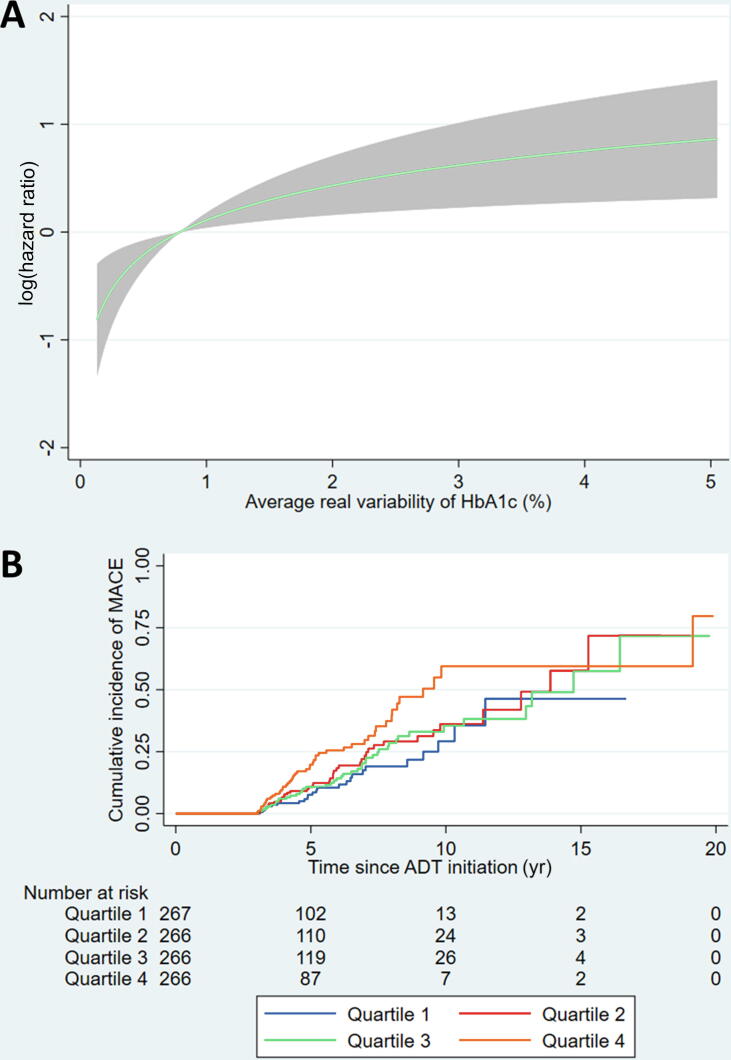
(A) Fractional polynomial plot showing the association between the average real variability (ARV) of HbA1c and the risk of major adverse cardiovascular events (MACEs) across the observed range of HbA1c ARV. (B) Kaplan-Meier curves showing the cumulative incidence of MACEs in patients in each quartile of the ARV of HbA1c. ADT = androgen deprivation therapy.

Over a median follow-up of 4.3 yr (2.8–6.7 yr), 159 patients (14.9%) had MACEs. Higher VVHV was associated with a higher risk of MACEs, as measured by both CV (adjusted hazard ratio [aHR]; per SD] 1.21 [95% confidence interval 1.02, 1.43], *p* = 0.029; Table 2 and Fig. 2A) and ARV (aHR [per SD] 1.25 [1.06, 1.48], *p* = 0.008; Table 2 and Fig. 3A); one SD of CV corresponded to 0.082 and one SD of ARV corresponded to 0.72%. When analysed as quartiles, patients in the highest quartile of both CV (aHR 1.69 [1.03, 2.77], *p* = 0.037; Table 2 and Fig. 2B) and ARV (aHR 1.90 [1.14, 3.16], *p* = 0.014; Table 2 and Fig. 3B) of HbA1c had a significantly higher risk of MACEs than those in the lowest quartile. However, among patients who had at least three HbA1c results within the 3 yr prior to ADT initiation, neither per-unit changes nor percentage changes in VVHV were significantly associated with the risk of MACEs (Supplementary Table 7).

**Table 2:** Results of Cox regression examining the associations between visit-to-visit HbA1c variability and the risk of major adverse cardiovascular events

Variability measure		Variability as continuous variable (per SD increase)		Variability as quartiles			
		Univariable	Multivariable a	Q1	Q2 a	Q3 a	Q4 a
CV	Median (IQR)	0.081 (0.046–0.135)		0.030 (0.020–0.039)	0.063 (0.054–0.072)	0.104 (0.092–0.116)	0.186 (0.157–0.239)
	HR (95% CI)	1.23 (1.06, 1.44), *p* = 0.008	1.21 (1.02, 1.43), *p* = 0.029	1 (reference)	0.85 (0.51, 1.42), *p* = 0.532	1.29 (0.80, 2.08), *p* = 0.306	1.69 (1.03, 2.77), *p* = 0.037
ARV	Median (IQR)	0.57 (0.31–1.03)		0.20 (0.15–0.27)	0.43 (0.38–0.50)	0.75 (0.65–0.85)	1.50 (1.20–2.05)
	HR (95% CI)	1.31 (1.12, 1.53), *p* = 0.001	1.25 (1.06, 1.48), *p* = 0.008	1 (reference)	1.25 (0.76, 2.08), *p* = 0.383	1.31 (0.80, 2.17), *p* = 0.283	1.90 (1.14, 3.16), *p* = 0.014

ADT = androgen deprivation therapy; ARV = average real variability; CI = confidence interval; CV = coefficient of variation; HR = hazard ratio; Q = quartile; SD = standard deviation.

a Adjusted for age, medical castration, bilateral orchiectomy, ADT duration, hypertension, atrial fibrillation, and baseline HbA1c.

### 3.3 Subgroup analysis of the prognostic value of VVHV

In the subgroup analysis by prior diagnosis of DM (Supplementary Table 8), higher VVHV, as measured by CV (aHR [per SD] 1.25 [1.03, 1.52], *p* = 0.024) and ARV (aHR [per SD] 1.27 [1.06, 1.52], *p*  =  0.009), was associated with a higher risk of MACEs in patients with a prior diagnosis of DM (*N* = 850), but not in those without (*N* = 215). There was no significant interaction between either CV (*p*_interaction_ = 0.396) or ARV (*p*_interaction_ = 0.603) of HbA1c and prior diagnosis of DM in terms of the risk of MACEs.

Similarly, in the subgroup analysis by prior use of any antidiabetic medication(s) (Supplementary Table 9), higher VVHV, as measured by CV (aHR [per SD] 1.23 [1.01, 1.50], *p* = 0.041) and ARV (aHR [per SD] 1.24 [1.04, 1.49], *p* = 0.020), was associated with a higher risk of MACEs in patients with the prior use of any antidiabetic medication(s) (*N* = 788), but not in those without (*N* = 277). There was no significant interaction between either CV (*p*_interaction_ = 0.583) or ARV (*p*_interaction_ = 0.972) of HbA1c and prior use of any antidiabetic medication(s) in terms of the risk of MACEs.

A subgroup analysis by the type of ADT found similar results (Supplementary Table 10), with higher VVHV, as measured by CV (aHR [per SD] 1.32 [1.05, 1.67], *p* = 0.017) and ARV (aHR [per SD] 1.31 [1.04, 1.65], *p* = 0.024), being associated with a higher risk of MACEs in patients who underwent only medical castration (*N* = 635), but not in those who underwent only BO (*N* = 303) or those who underwent both medical castration and BO (*N* = 127), as summarised in Supplementary Table 10. No significant interactions were found between the type of ADT and VVHV in terms of the risk of MACEs.

### 3.4 Sensitivity analysis of the prognostic value of VVHV

In total, 367 patients (34.5%) died without having MACEs. The *a priori* sensitivity analysis using multivariable Fine and Gray competing risk regression found that higher ARV of HbA1c was associated with a higher cumulative incidence of MACEs (adjusted subhazard ratio (per SD) 1.15 [1.01, 1.32], *p* = 0.037). However, CV of HbA1c was not significantly associated with the cumulative incidence of MACEs (adjusted subhazard ratio [per SD] 1.11 [0.96, 1.29], *p* = 0.142).

The post hoc sensitivity analysis using restricted mean survival time (Supplementary Table 11) showed consistent results, where patients in the highest quartile of both CV and ARV had significantly shorter restricted mean survival time than those in the lowest quartile.

The second post hoc sensitivity analysis, which analysed only patients with at least 1 yr of ADT (*N* = 1030), also showed consistently that increases in both CV (aHR [per SD] 1.21 [1.02, 1.44], *p* = 0.026) and ARV (aHR [per SD] 1.26 [1.07, 1.49], *p* = 0.006) of HbA1c were associated with an increased risk of MACEs.

## 4 Discussion

In this study, we showed that ADT may increase VVHV and that higher VVHV, but not changes in VVHV, was associated with a higher risk of MACEs among patients with PCa receiving ADT. To the best of the authors’ knowledge, this is the first study that explored the effects of ADT on VVHV, as well as the prognostic value of VVHV in the context of ADT.

We found that VVHV increased after ADT, consistent with prior findings of ADT being associated with poor glycaemic control [6], [7], [8], [9], [10]. Previous studies about glycaemic control in patients receiving ADT focused on time point–specific HbA1c or fasting glucose levels, which capture only a snapshot of a patient’s glycaemic metabolism and ignore temporal variations in glycaemic indices. VVHV adds a longitudinal element to the assessment of glycaemic control, with higher VVHV indicating lower glycaemic stability. Numerous measures of VVHV exist [12], [14], [25]. Here, we chose CV and ARV as measures of VVHV. CV is one of the most common measures of VVHV, as its definition (SD divided by mean) inherently considers the effects of mean HbA1c on VVHV. Meanwhile, ARV focuses on differences between consecutive measurements and has been found to be superior to SD in terms of prognostic significance [24]. Originally devised for blood pressure measurements, ARV has been adopted for VVHV [12] as well as for visit-to-visit fasting glucose variability [26], [27]. We showed that both CV and ARV of HbA1c increased after ADT initiation, providing robust evidence that ADT adversely affects glycaemic stability.

Our finding that higher VVHV was prognostic of MACEs agrees with prior studies of VVHV in other populations [11], [12], [13], [14], [15]. Notably, our results indicated that a threshold effect may exist in the relationship between VVHV and the risk of MACEs, as only the highest quartile, but not the second or third quartiles, was associated with an increased risk of MACEs compared with the lowest quartile. Clinically, this may necessitate determination of an upper limit of normal VVHV, rather than aiming to minimise VVHV; further, larger studies are required. Additionally, our subgroup analysis found no significant interaction between VVHV and prior diagnosis of DM, use of antidiabetic medications, or the type of ADT, suggesting that VVHV is prognostic regardless of these factors. Although associations were insignificant in several subgroups, the statistical insignificance was probably due to the small sample sizes. Nevertheless, competing risk regression found significant association only between HbA1c ARV and the risk of MACEs. Kim and colleagues [12] have made similar observations in patients with type 2 DM, possibly indicating that ARV is a more robust measure of VVHV and prognosticator.

### 4.1 Clinical relevance and future directions

Clinically, our findings reinforced the potential utility of VVHV as a tool for cardiovascular risk stratification. Little has been done in terms of cardiovascular risk stratification for patients with PCa receiving ADT. VVHV may be a simple marker that can be explored for such purposes. More generally, our findings should raise clinicians’ awareness of the importance of VVHV and prevent fluctuations in HbA1c from being dismissed as random or measurement errors.

Nonetheless, much work remains before VVHV may be adopted for clinical use. Having observed a possible threshold effect, normal values of VVHV need to be established for patients with PCa receiving ADT. Additionally, drivers of VVHV remain unclear: whilst medication adherence may be an intuitive driver, studies have demonstrated associations between VVHV, inflammation, and oxidative stress [28], [29]. Given the intimate relationships between inflammation and both cancer and cardiovascular diseases [30], [31], the association between VVHV and the risk of MACEs may vary depending on a patient’s inflammatory state. Lastly, whilst it is enticing to suggest VVHV to be a treatment target of glycaemic control, it remains unclear how interventions, both pharmacological and nonpharmacological, influence VVHV. Findings from our subgroup analysis suggested that the usage of antidiabetic medication(s) may be associated with smaller changes in VVHV as compared with nonusage. However, it remained unclear whether such an association was independent of confounders, and such findings should be viewed as hypothesis generating only. These areas require further investigation before VVHV may be utilised clinically.

### 4.2 Strengths and limitations

Utilising data from a population-based database, this study included as many patients as pragmatically possible from Hong Kong, increasing the representativeness of our findings. Additionally, we demonstrated robust associations between VVHV and the risk of MACEs in multiple subgroup and sensitivity analyses, reinforcing the validity of our findings. Nonetheless, this study has some limitations. First, many patients had fewer than three HbA1c levels recorded within the 3 yr after ADT initiation, with only 1065 of the 13 537 patients (7.9%) who fulfilled the inclusion criteria analysed, limiting the generalisability of our findings and necessitating larger studies to validate our findings. Furthermore, this study selected patients with high cardiometabolic risks, as they were more likely to receive frequent HbA1c testing than those with low metabolic risks. Therefore, it is unclear whether VVHV would be as prognostic in patients with lower metabolic risks. Similarly, our selection criteria for patients with at least 6 mo of ADT limited generalisability of our findings to patients receiving shorter durations of ADT. Future studies should therefore further explore the effects that shorter courses of ADT may have on VVHV, as well as the prognostic value of VVHV in these patients. Nonetheless, our findings were consistent with prior findings, including those in the general population [25], meaning that VVHV is likely prognostic in patients with lower metabolic risks as well.

Additionally, the observational nature of this study predisposed to residual and unmeasured confounders. Specifically, some studies have suggested that gonadotropin-releasing hormone agonists and antagonists may differ in the risk of MACEs [32], although this has remained highly controversial following the publication of the PRONOUNCE trial, the first randomised controlled trial specifically designed to compare the cardiovascular safety of gonadotropin-releasing hormone agonists and antagonists, which found no significant difference in the risk of MACEs between these agents [33]. Moreover, cancer staging, histology, disease risk profile, and individual indications for specific treatment regimens were not available. Nevertheless, we have considered many important cardiovascular risk factors for multivariable adjustment. Lastly, due to the deidentified nature of the database used (CDARS), the data could not be adjudicated individually, and miscoding of diagnoses and outcomes was possible. Nonetheless, all data inputs were performed by the patients’ treating clinicians who were independent of the authors, and none of the authors had the rights to alter recorded data. Previous studies of CDARS have also demonstrated good data completeness and coding accuracy [17].

## 5 Conclusions

In patients with PCa receiving ADT, VVHV increased after ADT initiation. Higher VVHV was associated with an increased risk of MACEs, independent of prior diagnosis of DM, use of antidiabetic medication(s), and type of ADT. Further studies are required to validate our findings and to further explore VVHV as a potential tool for cardiovascular risk stratification in patients with PCa receiving ADT.

  ***Author contributions*:** Gary Tse had full access to all the data in the study and takes responsibility for the integrity of the data and the accuracy of the data analysis.

  *Study concept and design*: Chan.

*Acquisition of data*: Lee, K. Liu, Hui, Tse, C.F. Ng.

*Analysis and interpretation of data*: Chan, Dee, K. Ng, Tse.

*Drafting of the manuscript*: Chan.

*Critical revision of the manuscript for important intellectual content*: Chan, Lee, K. Liu, Hui, Dee, K. Ng, Satti, T. Liu, Tse, C.F. Ng.

*Statistical analysis*: Chan.

*Obtaining funding*: T. Liu, Tse.

*Administrative, technical, or material support*: Lee, K. Liu, Dee, K. Ng, Satti, Tse, C.F. Ng.

*Supervision*: Dee, K. Ng, T. Liu, Tse, C.F. Ng.

*Other*: None.

  ***Financial disclosures:*** Gary Tse certifies that all conflicts of interest, including specific financial interests and relationships and affiliations relevant to the subject matter or materials discussed in the manuscript (eg, employment/affiliation, grants or funding, consultancies, honoraria, stock ownership or options, expert testimony, royalties, or patents filed, received, or pending), are the following: Edward Christopher Dee is funded in part through the Cancer Center Support Grant from the National Cancer Institute (P30 CA008748). All other authors have no conflict of interest to report.

  ***Funding/Support and role of the sponsor*:** This work was funded by the Tianjin Key Medical Discipline (Specialty) Construction Project (project number: TJYXZDXK-029A). The funder played no role in any part of this study.

  ***Data sharing*:** All data underlying this study are available on reasonable request to the corresponding authors.
