# Supplementary material for: HbA1c Variability and Cardiovascular Events in Patients with Prostate Cancer Receiving Androgen Deprivation Therapy
Source: Eur Urol Open Sci. 2022 Dec 15;47:3–11. doi: 10.1016/j.euros.2022.11.002 (PMC9806701; doi:10.1016/j.euros.2022.11.002)
Supplement: Supplementary Data 1 [file mmc1.pdf]

SUPPLEMENTARY MATERIAL

Supplementary methods

Covariate recorded

The following baseline covariates were recorded: age, type of androgen deprivation therapy (ADT), duration of ADT, comorbid conditions defined using ICD-9 codes listed in **Supplementary Table 1** (hypertension, diabetes mellitus (DM), dyslipidaemia, ischaemic heart disease, chronic kidney disease, atrial fibrillation, known malignancy), prior radiotherapy, prior radical prostatectomy, use of medications (angiotensin-converting enzyme inhibitors or angiotensin receptor blockers, androgen receptor signaling inhibitors, beta-blockers, metformin, sulphonylureas, insulins, dihydropyridine calcium channel blockers, antiplatelets, anticoagulants, chemotherapeutic agents, and steroids), and baseline HbA1c level.

Statistical analyses

Continuous variables were expressed as medians with interquartile ranges (IQRs). For patients who had at least three HbA1c test results available in the three years prior to ADT initiation, the CV, and ARV of HbA1c before and after ADT initiation were compared using the Wilcoxon signed rank test.

As the test for proportional hazards assumption based on Schoenfield's residuals showed no significant violation of the proportional hazards assumption, Cox proportional hazards models were used to evaluate the prognostic value of visit-to-visit HbA1c variability (VVHV) after ADT initiation, and the per-unit and percentage changes in VVHV. Exposure to medical and surgical castration were separately modelled as time-varying variables. Univariable Cox regression was performed for baseline variables to identify significant confounders (**Supplementary Table 3**; defined as p<0.10 on univariable Cox regression). These identified confounders were subsequently used for multivariable adjustment in multivariable Cox models with VVHV and changes in VVHV as continuous variables. Measures of VVHV were standardized, such that the results represent estimates per standard deviation (SD) increase in VVHV measures. Patients were then divided into quartiles by VVHV, and multivariable Cox models were fitted again with the first quartile as reference. Kaplan-Meier incidence curves were used to visualize the cumulative incidence of MACE over the study duration, and hazard ratios with 95% confidence intervals (CIs) were used as summary statistics. Furthermore, the HR across the observed spectrum of VVHV as compared

to the observed mean of VVHV was modelled and visualized using fractional polynomial curves.

Three *a priori* subgroup analyses were performed for both changes in VVHV and the prognostic value of VVHV. To better understand if the prognostic value of VVHV differ between diabetic and non-diabetic patients, a subgroup analysis was performed with stratification by known diagnosis of diabetes mellitus. Similarly, a subgroup analysis was performed with stratification by the use of any antidiabetic medication, with testing for interaction. Another subgroup analysis was performed for the type of ADT (medical castration, BO, or both) to understand in greater detail the prognostic power of VVHV in different types of ADT. Per-unit and percentage changes in the markers of VVHV were compared between subgroups using Mann-Whitney test or Kruskal-Wallis test as appropriate, while interaction terms were used in multivariable Cox regression to compare the prognostic value of the markers of VVHV between subgroups.

Three sensitivity analyses were performed. As non-cardiovascular mortality prohibits the observation of any potential major adverse cardiovascular event (MACE) in those who had not experienced MACE, non-cardiovascular mortality constitutes a competing event for MACE. Therefore, an *a priori* sensitivity analysis was performed using multivariable competing risk regression under the Fine and Gray sub-distribution model, with non-cardiovascular mortality as the competing event and the same adjusting variables as in the fully adjusted Cox model; sub-hazard ratios with 95% CIs were used as summary statistics.

Additionally, as the cumulative incidence curves crossed each other, a post hoc sensitivity analysis was performed where differences in restricted mean survival time were used to compare between groups. This approach does not rely on the proportional hazards assumption[1].

Finally, to reduce heterogeneity in the duration of androgen deprivation therapy, a second post hoc sensitivity analysis was performed where only patients with at least 18 months of androgen deprivation therapy were analyzed. This was also done in an effort to mitigate the herein lack of staging and disease risk profile data, as androgen deprivation therapy of one year or longer is unlikely to be used in patients with low-risk prostate cancer[2,3].

All p-values were two-sided, with $p<0.05$ considered statistically significant. All analyses were performed on Stata v16.1 (StataCorpLLC, College Station, Texas, USA).

Supplementary Table 1. International Classification of Diseases, Ninth Revision (ICD-9) codes used to identify outcomes and co-morbidities. All hereby listed codes include the corresponding sub-codes.

Prostate cancer	185													
Heart failure	428													
Myocardial infarction	410													
Diabetes mellitus	250													
Hypertension	401	402	403	404	405	437.2								
Atrial fibrillation	427.31													
Stroke	430	431	432	433	434	435								
Chronic obstructive pulmonary disease	490	491	492	496.0										
Ischaemic heart disease	410	411	412	413	414									
Chronic kidney disease	582	585	586											
Chronic liver disease	456.0	456.1	456.20	456.21	571	572.2	572.3	572.4	572.5	572.6	572.7	572.8		
Anaemia	280	281	282	283	284.0	284.1	284.8	284.9	285					
Dyslipidaemia	272.0	272.1	272.2	272.3	272.4									
Malignancy	140	141	142	143	144	145	146	147	148	149	150	151	152	153
		154	155	156	157	158	159	160	161	162	163	164	165	170
		171	172	173	174	175	179	179	180	181	182	183	184	185
		186	187	188	189	190	191	192	193	194	195	196	197	198
		199	200	201	202	203	204	205	206	207	208	209.0	209.1	209.2
		209.3												

Supplementary Table 2. International Classification of Diseases, Ninth Revision (ICD-9) and Tenth Revision (ICD-10) codes used to identify the cause of death. All hereby listed codes include the corresponding sub-codes.

Type of mortality	ICD codes
Cardiovascular mortality	ICD-9: 390-438<br>ICD-10: I00-I79

Supplementary Table 3. Results of univariable Cox regression.

	Hazard ratio [95% confidence interval]	p value
Age (years)	1.05 [1.02, 1.07]	<0.001*
Medical castration	0.62 [0.46, 0.85]	0.003*
Bilateral orchiectomy	1.49 [1.09, 2.04]	0.014*
ADT duration (years)	0.94 [0.90, 0.98]	0.002*
Hypertension	1.34 [0.98, 1.84]	0.070*
Diabetes mellitus	1.35 [0.83, 2.18]	0.224
Dyslipidaemia	1.07 [0.65, 1.75]	0.801
Ischaemic heart disease	1.03 [0.66, 1.61]	0.887
Chronic kidney disease	1.18 [0.48, 2.88]	0.713
Atrial fibrillation	2.36 [1.10, 5.06]	0.027*
Known malignancy	1.15 [0.65, 2.03]	0.627
Prior radiotherapy	1.10 [0.78, 1.56]	0.574
Prior radical prostatectomy	1.04 [0.49, 2.21]	0.927
ACEI/ARB use	0.90 [0.66, 1.23]	0.515
Beta-blocker use	1.23 [0.90, 1.69]	0.185
Metformin use	1.16 [0.83, 1.63]	0.373
Sulphonylurea use	1.02 [0.74, 1.41]	0.893
Insulin use	1.24 [0.81, 1.90]	0.327
Dihydropyridine CCB use	1.12 [0.82, 1.53]	0.485
Antiplatelet use	1.16 [0.83, 1.64]	0.380
Anticoagulant use	0.29 [0.04, 2.09]	0.221
Chemo use	3.14 [0.44, 22.57]	0.255
Steroid use	0.89 [0.57, 1.40]	0.625
Ever used ARSI	0.78 [0.56, 1.08]	0.132
Baseline HbA1c	1.13 [1.01, 1.27]	0.031*

ACEI, angiotensin-converting enzyme inhibitor. ADT, androgen deprivation therapy. ARB, angiotensin receptor blocker. ARSI, androgen receptor signaling inhibitor. CCB, calcium channel blocker.

* Baseline variables with p<0.10 which were then used in multivariable Cox regression for adjustment

Supplementary Table 4. Comparison of changes in visit-to-visit HbA1c variability between patients with and without diabetes mellitus. Medians and interquartile ranges are shown.

	With diabetes mellitus (N=655)	Without diabetes mellitus (N=54)	p value
Per-unit change in CV	0.024 [-0.015-0.073]	0.014 [0.002-0.030]	0.521
Percentage change in CV, %	41.0 [-19.0-146.9]	53.4 [51.2-153.4]	0.355
Per-unit change in ARV, %	0.183 [-0.100-0.526]	0.099 [-0.030-0.255]	0.201
Percentage change in ARV, %	40.0 -180.2-137.0]	58.7 [-22.2-156.4]	0.528

ARV, average real variability. CV, coefficient of variation.

Supplementary Table 5. Comparison of changes in visit-to-visit HbA1c variability between patients with and without use of antidiabetic medication(s). Medians and interquartile ranges are shown.

	With use of antidiabetic medication(s) (N=610)	Without use of any antidiabetic medication(s) (N=99)	p value
Per-unit change in CV	0.024 [-0.017-0.073]	0.020 [0.001-0.053]	0.659
Percentage change in CV, %	38.8 [-20.7-137.2]	55.7 [38.1-197.9]	0.025
Per-unit change in ARV, %	0.181 [-0.111-0.520]	0.117 [-0.015-0.335]	0.838
Percentage change in ARV, %	38.5 [-19.1-129.9]	61.5 [-8.3-184.5]	0.072

ARV, average real variability. CV, coefficient of variation.

Supplementary Table 6. Comparison of changes in visit-to-visit HbA1c variability between types of androgen deprivation therapy. Medians and interquartile ranges are shown.

	Medical castration only (N=217)	Bilateral orchiectomy only (N=189)	Both medical castration and bilateral orchiectomy (N=88)	p value
Per-unit change in CV	0.023 [-0.009-0.068]	0.023 [-0.028-0.076]	0.020 [-0.011-0.069]	0.907
Percentage change in CV, %	48.0 [-12.4-139.5]	38.5 [-33.2-149.6]	28.9 [-17.4-154.3]	0.606
Per-unit change in ARV, %	0.175 [-0.060-0.493]	0.175 [-0.159-0.514]	0.119 [-0.140-0.500]	0.799
Percentage change in ARV, %	48.9 [-12.6-133.3]	25.7 [-25.0-136.7]	42.6 [-24.0-147.9]	0.346

ARV, average real variability. CV, coefficient of variation.

Supplementary Table 7. Cox regression results for changes in visit-to-visit HbA1c variability.

	Univariable hazard ratio [95% confidence interval]	Multivariable hazard ratio [95% confidence interval] [1]
Per-unit change in CV	5.85 [0.44, 78.62], p=0.182	6.29 [0.45, 87.20], p=0.170
Percentage change in CV (per 10%)	1.00 [0.99, 1.01], p=0.796	1.00 [0.99, 1.01], p=0.694
Per-unit change in ARV	1.26 [0.92, 1.71], p=0.146	1.23 [0.91, 1.67], p=0.181
Percentage change in ARV (per 10%)	1.00 [1.00, 1.01], p=0.635	1.00 [1.00, 1.01], p=0.513

ARV, average real variability. CV, coefficient of variation.

[1] Adjusted for age, medical castration, bilateral orchiectomy, ADT duration, hypertension, atrial fibrillation, and baseline HbA1c.

Supplementary Table 8. Results of subgroup analysis by prior diagnosis of diabetes mellitus. Hazard ratios and the corresponding 95% confidence intervals are shown, with adjustment for age, medical castration, bilateral orchiectomy, ADT duration, hypertension, atrial fibrillation, and baseline HbA1c.

	With diabetes mellitus (N=850)	Without diabetes mellitus (N=215)	$p_{interaction}$
CV of HbA1c (per SD)	1.25 [1.03, 1.52], p=0.024	1.25 [0.81, 1.94], p=0.318	0.396
ARV of HbA1c (per SD)	1.27 [1.06, 1.52], p=0.009	1.33 [0.73, 2.41], p=0.353	0.603

ARV, average real variability. CV, coefficient of variation.

Supplementary Table 9. Results of subgroup analysis by baseline use of antidiabetic medication(s). Hazard ratios and the corresponding 95% confidence intervals are shown, with adjustment for age, medical castration, bilateral orchiectomy, ADT duration, hypertension, atrial fibrillation, and baseline HbA1c.

	With use of antidiabetic medication(s) (N=788)	Without use of any antidiabetic medication(s) (N=277)	$p_{interaction}$
CV of HbA1c (per SD)	1.23 [1.01, 1.50], p=0.041	1.26 [0.88, 1.81], p=0.207	0.583
ARV of HbA1c (per SD)	1.24 [1.04, 1.49], p=0.020	1.45 [0.92, 2.29], p=0.113	0.972

ARV, average real variability. CV, coefficient of variation.

Supplementary Table 10. Results of subgroup analysis by the type of androgen deprivation therapy. Hazard ratios and the corresponding 95% confidence intervals are shown, with adjustment for age, bilateral orchiectomy (for patients who had both medical castration and bilateral orchiectomy), ADT duration, hypertension, atrial fibrillation, and baseline HbA1c.

	Medical castration only (N=635)	Bilateral orchiectomy only (N=303)	Both medical castration and bilateral orchiectomy (N=127)
CV of HbA1c (per SD)	1.32 [1.05, 1.67], p=0.017	1.09 [0.80, 1.49], p=0.585 [1]	1.23 [0.80, 1.89], p=0.338 [3]
ARV of HbA1c (per SD)	1.31 [1.04, 1.65], p=0.024	1.22 [0.93, 1.60], p=0.152 [2]	1.32 [0.73, 2.39], p=0.352 [4]

ARV, average real variability. CV, coefficient of variation.
[1] $p_{interaction}$=0.351 with medical castration subgroup as reference
[2] $p_{interaction}$=0.623 with medical castration subgroup as reference
[3] $p_{interaction}$=0.401 with medical castration subgroup as reference
[4] $p_{interaction}$=0.497 with medical castration subgroup as reference

Supplementary table 11. Results of the post hoc sensitivity analysis using differences in restricted mean survival time to compare patients in each quartile of the coefficient of variation (CV) and average real variability (ARV) of HbA1c. Restricted mean survival time (in years) and the corresponding 95% confidence intervals are shown.

	Quartile 1	Quartile 2	Quartile 3	Quartile 4
CV of HbA1c	10.81 [9.94, 11.67] (reference)	11.03 [10.36, 11.70], p=0.690	10.23 [9.56, 10.91], p=0.307	9.39 [8.43, 10.35], p=0.031
ARV of HbA1c	10.00 [9.47, 10.52] (reference)	9.58 [9.06, 10.10], p=0.271	9.63 [9.14, 10.12], p=0.317	8.54 [7.90, 9.18], p=0.001

References for supplementary material

[1] Royston P, Parmar MKB. Restricted mean survival time: An alternative to the hazard ratio for the design and analysis of randomized trials with a time-to-event outcome. BMC Med Res Methodol 2013;13:1–15. https://doi.org/10.1186/1471-2288-13-152/FIGURES/3.

[2] Mohler JL, Antonarakis ES, Armstrong AJ, D’Amico A V., Davis BJ, Dorff T, et al. Prostate Cancer, Version 2.2019, NCCN Clinical Practice Guidelines in Oncology. J Natl Compr Canc Netw 2019;17:479–505. https://doi.org/10.6004/JNCCN.2019.0023.

[3] Cornford P, van den Bergh RCN, Briers E, Van den Broeck T, Cumberbatch MG, De Santis M, et al. EAU-EANM-ESTRO-ESUR-SIOG Guidelines on Prostate Cancer. Part II—2020 Update: Treatment of Relapsing and Metastatic Prostate Cancer. Eur Urol 2021;79:263–82. https://doi.org/10.1016/J.EURURO.2020.09.046.
